# The Superiority of Kosso as a Remedy for the Tape Worm

**Published:** 1866-04

**Authors:** 


*The Superiority of Kosso as a Remedy for the Tape Worm.*—From the Buffalo *Medical and Surgical Journal* we make the following extract from the proceedings of the Buffalo Medical Association:

"Dr. C. L. Dayton presented two specimens of tape worm, and believed that the head had in both cases been expelled with the body, but had not made a microscopic examination. The first patient was a man of middle age, and the second a young lady of eighteen. Gave ℥ i of kosso in ʒ i doses every four hours in each case.

Dr. Cronyn remarked that the case related by Dr. Dayton brought to mind the superior value of the kosso. Had lately given it to a patient who had taken other remedies in vain. The patient soon passed thirty feet of tape worm, and a few days after resort was again had to the remedy, with the result of the passage of about one hundred feet.

Dr. Dayton finds that kosso often causes vomiting unless preceded by a cathartic, the operation of which will almost invariably prevent vomiting.

Dr. Miner thinks that kosso is usually given in too large doses, and believes that half the usual quantity is sufficient."

